# Traffic-driven ions motion optimization-based clustering routing protocol for cognitive radio sensor networks

**DOI:** 10.1371/journal.pone.0272505

**Published:** 2022-09-01

**Authors:** Jihong Wang, Hao Ni, Yiyang Ge, Shuo Li

**Affiliations:** School of Electrical Engineering, Northeast Electric Power University, Jilin, Jilin, China; Jinan University, China, HONG KONG

## Abstract

In cognitive radio sensor networks, single clustering protocol cannot simultaneously satisfy the various requirements of time-triggered and event-driven traffic, as a result, different kinds of clustering protocols are designed to serve them separately. In addition, for event-driven traffic, the long delay incurred by clustering and searching for available routes after events results in poor timeliness of information transmission. In order to solve above problems, a traffic-driven ions motion optimization-based clustering routing protocol (TD-IMOCRP) is proposed in this paper. For the first time, time-triggered and event-driven traffic can be served by a single clustering protocol. To be specific, ions motion optimization algorithm is leveraged to automatically determine the optimal number of clusters and form basic clustering structure. In this case, time-triggered traffic can be periodically served. Priority-based schedule and corresponding frame structure are designed to ensure priority delivery of event-driven information. The clustering architecture built for time-triggered traffic is leveraged, and there is no cluster construction and route selection after emergent events. Only the CRSNs nodes which discover emergent events and corresponding CHs participate in data transmission, which means that TD-IMOCRP covers fewer nodes, especially when the sink is located at the corner. Therefore, it can help reduce node energy consumption and delay. Simulation results demonstrate that compared with representative event-driven clustering protocols, TD-IMOCRP can decrease the average number of covered nodes and the total energy consumption by more than 66.3% and 25%, respectively. In addition, when serving time-triggered traffic, TD-IMOCRP can achieve almost the same performance as its basic version IMOCRP which is better than majority of current time-triggered clustering protocols. In a word, TD-IMOCRP can guarantee in-time delivery of event-driven information while guaranteeing its performance of serving time-triggered traffic.

## 1. Introduction

In cognitive radio sensor networks (CRSNs), cognitive radio technology enables CRSNs nodes to dynamically access licensed spectrum for communication without affecting primary users (PUs), which dramatically improves spectrum efficiency and network surveillance capability [1, 2]. In order to improve energy efficiency, CRSNs nodes are usually grouped into different clusters by using certain clustering protocols. Information from cluster members (CMs) is aggregated and fused at their cluster heads (CHs) and then relayed to the sink [3]. Clustering protocols for CRSNs have the advantages of improving network connectivity and scalability, reducing information transmission delay and energy consumption [4]. Therefore, they are widely concerned by academia and industry.

Most existing clustering protocols for CRSNs are designed specifically for a particular type of traffic, i.e., time-triggered traffic or event-driven traffic, and they cannot satisfy the distinguished requirements of different traffic types simultaneously. Therefore, in order to serve different traffic types, each CRSNs node should run multiple clustering protocols, which poses a big challenge to the resource-constrained CRSNs nodes.

1. Time-triggered clustering protocols periodically construct clusters to transmit the surveillance information to the sink. For a typical time-triggered clustering protocol, there are 3 problems which need to be solved [5]: What is the optimal number of clusters? How to select CHs? How to form clusters? Our previous work in [6] applies swarm intelligence algorithm, i.e., ions motion optimization (IMO) algorithm to determine the optimal number of clusters and CHs automatically while minimizing the total control overhead incurred during clustering process. Ions motion optimization-based clustering routing protocol (IMOCRP) is proposed and the simulation results show that IMOCRP gains obvious advantages over existing time-triggered clustering protocols proposed for CRSNs in terms of network lifetime and network surveillance capability. However, IMOCRP cannot solve the in-time delivery of emergent event information which may have a large impact on network operation.
2. Event-driven clustering protocols form clusters in the corridor between the certain event and the sink after the occurrence of the event, and these clusters are only maintained until the end of the event. For a typical event-driven clustering protocol, there are also 3 problems to be solved: How to select eligible nodes? How to select CHs among these eligible nodes? How to form clusters? Current event-driven clustering protocols usually determine eligible nodes among those which are located in the corridor between the event and the sink according to some predetermined conditions. Therefore, many CRSNs nodes will be involved in the event information transmission, which will result in unnecessary energy waste and longer information transmission delay.

To the best of our knowledge, there is no universal clustering protocol for CRSNs which can serve the time-triggered traffic and event-driven traffic simultaneously. In order to solve above problems and guarantee the in-time delivery of event-driven information, a traffic-driven IMOCRP (TD-IMOCRP) is proposed in this paper based on IMOCRP. The contributions of our TD-IMOCRP can be summarized as follows:

1. TD-IMOCRP is the first universal clustering protocol specifically designed for CRSNs which can satisfy the distinguished requirements of both traffic types. In order to ensure the optimal network performance when serving time-triggered traffic, IMOCRP is leveraged to group nodes into clusters periodically with the purpose of minimizing the average node energy consumption and the standard deviation of node residual energy. Above clustering architecture is directly adopted by event-driven traffic to avoid the extra cluster construction and route selection after emergent events, which can help dramatically reduce latency.
2. Higher priority is further given to event-driven traffic by designing special *Fr* frame structure to guarantee the in-time delivery of event information. Only the CRSNs nodes which discover emergent events and corresponding CHs participate in the event-driven information transmission. Fewer nodes are involved, which helps further reduce node energy consumption and delay. Simulation results show that TD-IMOCRP can achieve the goals of periodical network surveillance and emergency monitoring simultaneously.

The rest of this paper is organized as follows. Section 2 reviews related works and compares existing clustering protocols designed for CRSNs to illustrate the advantages of TD-IMORCR from qualitative aspects. Section 3 illustrates the detailed design principles of TD-IMOCRP. Section 4 provides simulation results and corresponding discussion to verify the effectiveness of TD-IMOCRP. Section 5 concludes the paper and indicates our future research directions.

## 2. Related works

Based on data report model, current clustering protocols for CRSNs can roughly be divided into two types, that is, time-triggered clustering protocols and event-driven clustering protocols [7]. Time-triggered clustering protocols periodically group CRSNs nodes into clusters, i.e., the protocol operation lasts from network deployment to the end of network lifetime. They are suitable for continuous and periodical information collection. Event-driven clustering protocols are triggered by emergent events, and clusters disappear immediately after events end up. They do not maintain clusters by periodical calculation and communication, which is more energy-efficient [8]. Next, we will review existing time-triggered clustering protocols and event-driven clustering protocols for CRSNs and the comparison results among them are shown in Table 1 below.

**Table 1:** Characteristics analysis of existing clustering protocols for CRSNs.

References	Traffic type	Control overhead	Objective	Disadvantages
[9]	time-triggered	2*N*×*ite*_*DSAC*_	MinDC	cannot guarantee in-time delivery of emergent event information
[11]	time-triggered	3*N*×*ite*_*EACRP*_	×	
[12]	time-triggered	*N*	MinSSD	
[13]	time-triggered	3*N*	MinDM	
[14]	time-triggered	2*N*	×	
[18]	time-triggered	—	×	
[19]	time-triggered	3*N*-*K*	×	
[20]	time-triggered	*N*	MinASDE	
[21]	time-triggered	4*N*	×	
[22]	time-triggered	2*N*+2*N*_*candi*_	×	
[23]	time-triggered	*N+2N*_*candi*_	×	
[6]	time-triggered	*N*	MinASDE	
[24]	event-driven	*N*_*candieli*_+3*N*_*eli*_	×	increase node energy consumption and delay
[25]	event-driven	*N*_*candieli*_+3*N*_*eli*_	×	
[26]	event-driven	>*N*_*candieli*_+3*N*_*eli*_	×	

Note: *N* is the number of living nodes in current round; *ite*_*DSAC*_ and *ite*_*EACRP*_ are the number of merging iterations performed by DSAC and EACRP, respectively;—represents that the corresponding value is unable to be explicitly quantified; *K* is the optimal number of CHs; *N*_*candi*_ is the number of candidate CHs in uneven clustering protocols; *N*_*candieli*_ is the number of candidate eligible nodes while *N*_*eli*_ is the number of final eligible nodes; MinDC: minimize the total energy consumption of data communication, MinSSD: minimize the sum of squares of distance from CMs to cluster center, MinDM: minimize the distortion in multimedia quality incurred by packet losses and latency, MinASDE: minimize the average node energy consumption and the standard deviation of node residual energy; “×” denotes the corresponding problem has not been solved.

1. Time-triggered clustering protocols for CRSNs
  Most clustering protocols for CRSNs are time-triggered clustering protocols. The optimal number of clusters in these protocols is theoretically derived with various purposes, for example, minimizing the total energy consumption of data communication [9–11], minimizing the sum of squares of distance from CMs to cluster center [12] or minimizing the quality distortion introduced by packet losses and delay [13]. Distributed clustering protocols determine CHs and construct clusters by local information exchange. For example, cognitive low energy adaptive clustering hierarchy (CogLEACH) [14] which is a probabilistic clustering algorithm is proposed specifically for CRSNs by improving legacy low energy adaptive clustering hierarchy (LEACH) protocols [15–17]. Each CRSNs node can decide whether itself can become a CH or not by comparing its CHs probability with a random number; Distributed network stability-aware clustering protocol in [18] integrates spectrum dynamics and energy consumption for the first time, and cluster formation is repeated by excluding the maximum-weight CH and its neighbors until all CRSNs nodes are clustered. In centralized clustering protocols [19–21], the sink receives necessary information from CRSNs nodes, selects final CHs by using K-nearest neighbor algorithm or intelligent algorithms and then informs CRSNs nodes about the clustering results. Different from above uniform clustering protocols, uneven clustering protocols [22, 23] group CRSNs nodes into clusters with different sizes. In other words, cluster radius increases as the distance from the sink increases, which can help balance the residual energy among CHs. In our previous work [6], IMOCRP is proposed which aims at minimizing the average node energy consumption and the standard deviation of node residual energy. It can determine CHs and clustering relationship automatically. There is no need to compete for CHs and theoretically derive the optimal number of clusters. Therefore, IMOCRP can well adapt to dynamic characteristics of CRSNs and prolong network lifetime.
  However, all time-triggered clustering protocols are designed for periodical network surveillance and cannot guarantee in-time delivery of emergent event information which may have a large impact on network operation.
2. Event-driven clustering protocols for CRSNs
  Event-to-sink spectrum-aware clustering protocol (ESAC) in [24] builds temporary clusters between the event area and the sink only after occurrence of a certain event. Determining factors such as available channels and Euclidean distance are considered for CHs selection. On the basis of ESAC, mobility-aware ESAC (mESAC) protocol in [25] takes mobile nodes into consideration and it can improve inter-cluster connectivity. Energy-aware event-driven routing protocol (ERP) in [26] is proposed by improving channel selection scheme of ESAC, and the channel with low occupancy probability is chosen as cluster channel. However, in event-driven clustering protocols, CHs selection and route selection are both triggered after the event, which will increase the information transmission delay and significantly affect the timeliness of event handling.
  In order to conquer the limitations of current time-triggered and event-driven clustering protocols and ease the burden of resource-constrained CRSNs nodes, a universal clustering protocol TD-IMOCRP is specifically designed for CRSNs on the basis of IMOCRP. For time-triggered traffic, the optimal number of clusters and the identity of CHs are automatically determined by leveraging the excellent characteristics of IMO. TD-IMOCRP gives higher priority to event-driven traffic to guarantee reasonable clustering and in-time information transmission. To be specific, the clustering architecture built for time-triggered traffic is directly leveraged, and there is no cluster construction and route selection after emergent events. Only the CRSNs nodes which discover the emergent events and the corresponding CHs participate in data transmission. Consequently, TD-IMOCRP covers fewer nodes to transmit the event-driven information, especially when the sink is located at the corner. Therefore, it can help reduce node energy consumption and delay.

## 3. Traffic-driven ions motion optimization-based clustering routing protocol design for CRSNs

### 3.1 System model

The system model is composed of 3 parts, that is, network model, spectrum usage model of PUs and energy consumption model. The network model describes the size and the composition of the network, the functions of CRSNs nodes and the traffic characteristics. As the channel availability at each CRSNs node is heavily affected by spectrum usage of PUs, we adopt Semi-Markov process to imitate the dynamic behaviors of PUs, and how the model works is described in the next subsection. Remaining energy is a precious resource for CRSNs nodes, when it drops to 0, CRSNs nodes cannot function anymore, and they will die. Therefore, it is important to quantify the energy consumption of information transmission and reception which determines node residual energy, and the details are presented in the third subsection.

#### 3.1.1 Network model

**Fig 1:**
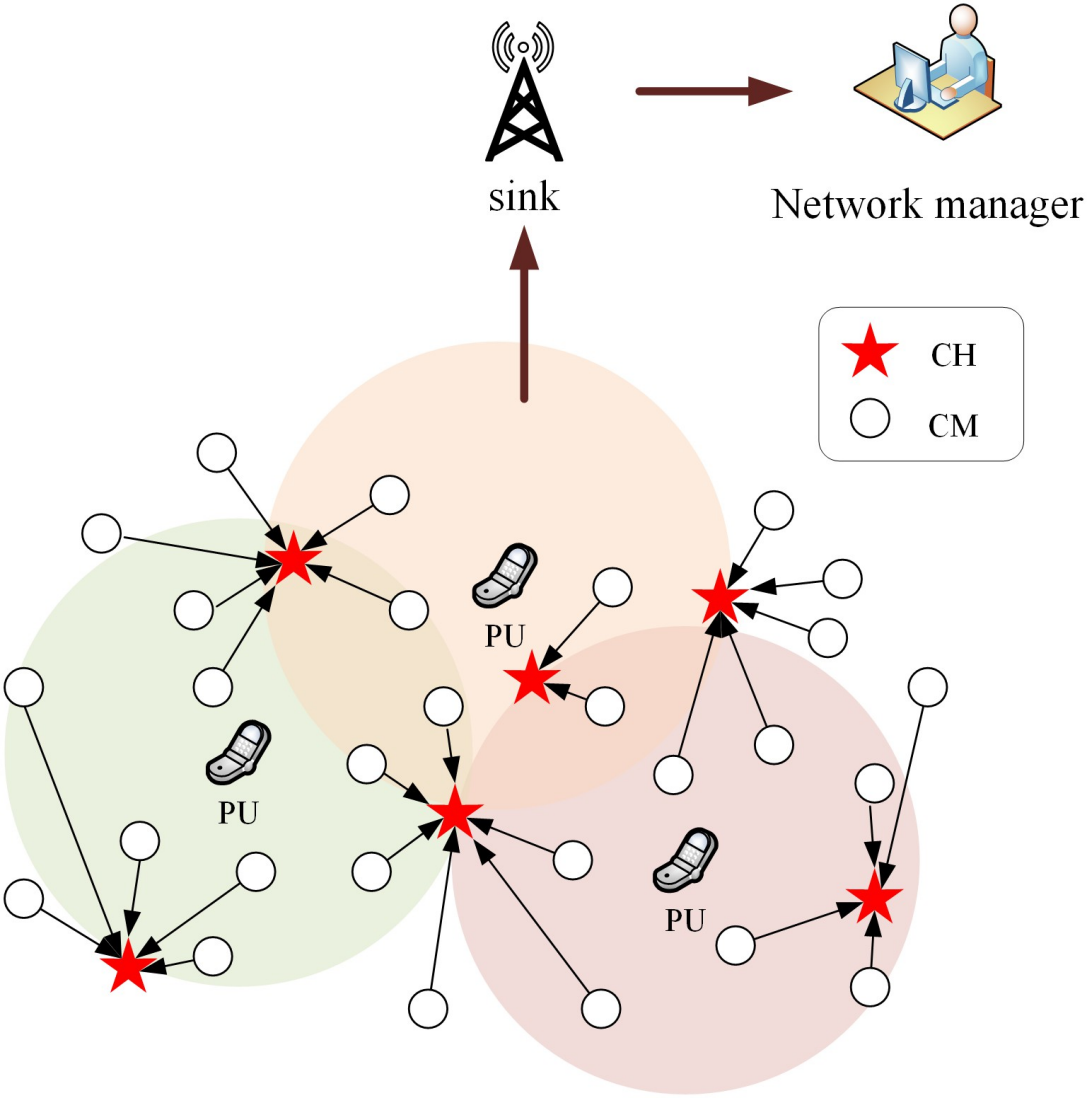
Illustration of CRSNs clustering structure.

Assuming that *N* homogeneous CRSNs nodes and *P* PUs are randomly distributed in an 100m×100m area, and the sink is located at the center of the network (or at position (0,0)). The detailed network structure is shown in Fig 1. The whole network is static, which means that each CRSNs node will not change its position once deployed. Each CRSNs node *i* can acquire its residual energy *E*(*i*)_*residual*_*t*_ and geographical location (*x*_*i*_,*y*_*i*_), and it can also exchange control information with its neighbors through common control channel (CCC) which is available to all CRSNs nodes. Each CRSNs node can perform accurate spectrum sensing, and sensing delay and errors are neglected. CRSNs nodes sense behaviors of dynamic PUs and detect idle spectrum to transmit information through clustering structure. Each CM periodically sends detected time-triggered data to its CH. The CH compresses and aggregates its own data with the data received from all its members, and then it relays the aggregated data to the sink. Finally, the monitored information is sent to network manager. In this paper, perfect aggregation [27] is assumed at CHs, which means that each CH can aggregate all data from its members into a single packet with fixed length, and detailed aggregation algorithm is not in the scope of this paper. Emergent events may happen at any time any place, and the information about these events should be delivered to the sink as soon as possible, as they will have a large impact on network operation. Therefore, event-driven data is given higher priority over time-triggered data.

#### 3.1.2 Spectrum usage model of PUs

Semi-Markov process is utilized to imitate dynamic behaviors of PUs [28, 29]. PUs’ signal on each licensed channel dynamically alternates between ON and OFF states, and the time length of each state is an independent random variable. CRSNs nodes which are located within the interference protection range of each PU monitor the channel. If PU is in ON state, CRSNs nodes cannot use this channel at present, otherwise, CRSNs nodes can have the opportunity to access the idle licensed channel and transmit data.

**Fig 2:**
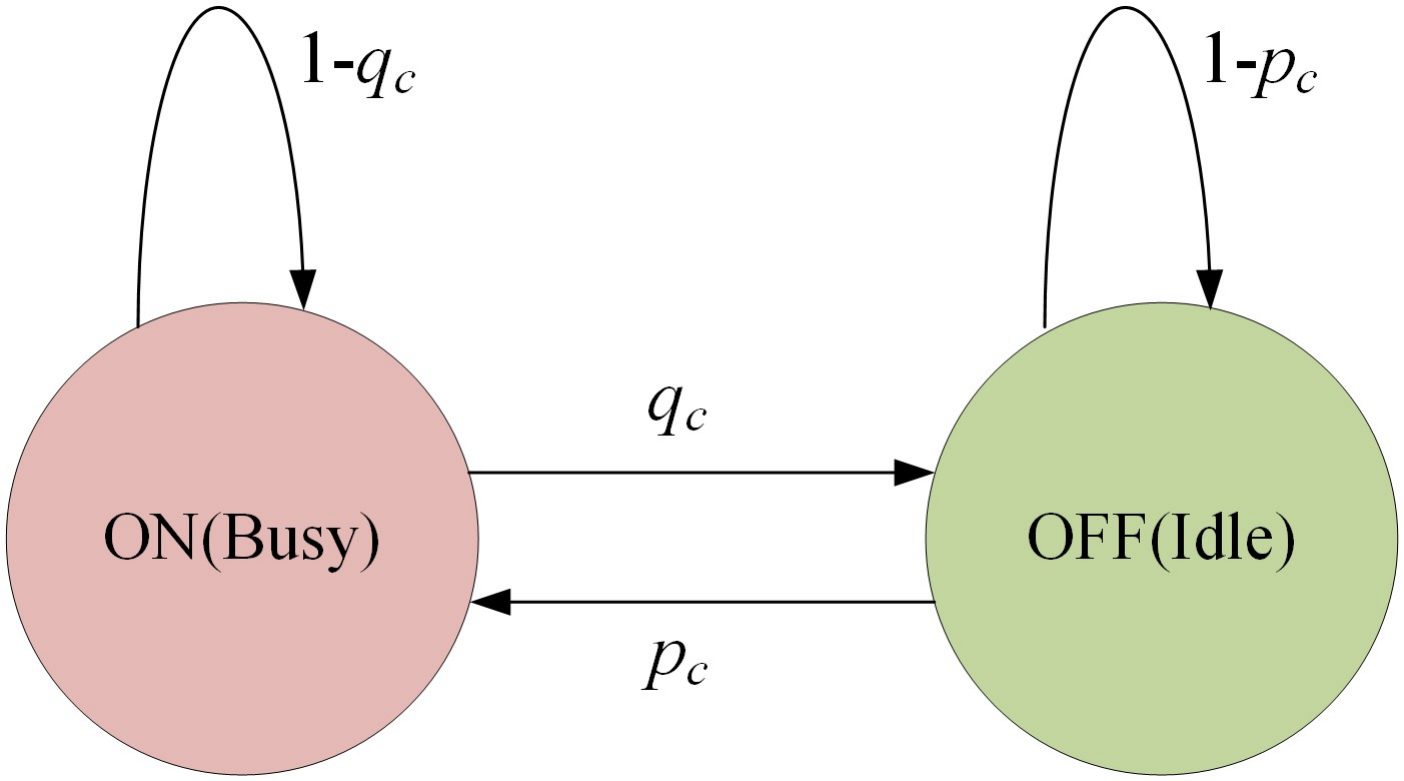
ON/OFF model for spectrum usage of PUs.

As shown in Fig 2, *p*_*c*_ denotes the probability of changing from OFF state to ON state, while *q*_*c*_ represents the transition probability in the opposite direction. In this paper, idle probability of each PU is assumed to be fixed and the same [14].

#### 3.1.3 Energy consumption model

The classic energy consumption model for wireless communication [15] is applied to calculate the energy consumption of CRSNs nodes. If a source node sends *l* bits of data to its destination *d* meters away, the energy consumption of the source node is denoted by Eq (1):

$$E_{trans}=\left\{\begin{array}{l} l\times E_{elec}+l\times E_{fs}\times d^{2}\;if\;d\leq d_{0}\\ l\times E_{elec}+l\times E_{mp}\times d^{4}\;otherwise \end{array}\right.$$ (1)

where *E*_*trans*_ is composed of 2 parts, that is, the energy consumption of electronic circuits and amplifier fading. Electronics energy per bit *E*_*elec*_ is decided by factors such as digital coding, modulation, filtering and signal transmission. Energy consumption of amplifier fading is related to distance. If *d*≤*d*_0_, the energy consumption of data transmission obeys free-space loss model, and it is proportional to *d*^2^. Otherwise, the energy consumption of data transmission follows multi-path loss model, and it is proportional to *d*^4^. The corresponding coefficients in these two cases are denoted by *E*_*fs*_ and *E*_*mp*_, respectively. In this paper, *E*_*elec*_ = 50nJ/bit, *E*_*fs*_ = 10pJ/bit/m^2^, *E*_*mp*_ = 0.0013pJ/bit/m^4^, *d*_0_ = 87.7m. Energy cost of receiving data at the destination is proportional to data packet size *l*, and it can be computed according to Eq (2).


$$E_{receive}=E_{elec}\times l$$ (2)

### 3.2 Objective function of IMO-based clustering

TD-IMOCRP can be applied to both types of traffic. For time-triggered traffic, based on IMOCRP, TD-IMOCRP periodically groups all living CRSNs nodes into clusters with fixed time interval, which means that the operation of TD-IMOCRP is based on rounds. The purpose of clustering is minimizing the average energy consumption and standard deviation of residual energy among nodes, which is shown in Eq (3).

$$OBJ=\frac{1}{AVG\left(E_{dissipate\_t}\right)+\alpha \times STD\left(E_{residual\_t}\right)}$$ (3)

where *AVG*(*E*_*dissipate*_*t*_) is the average energy consumption of all living nodes in round *t*, and it can be calculated by summing up the energy consumption of all living CRSNs nodes and then divided by their numbers, as shown in Eq (4); *STD*(*E*_*residual*_*t*_) is the standard deviation of residual energy among all living CRSNs nodes in round *t*, which is calculated by using Eq (5). *α* is model parameter which is utilized to adjust the relative weight of *STD*(*E*_*residual*_*t*_) in the objective function.

$$AVG\left(E_{dissipate\_t}\right)=\left(\sum_{n=1}^{N_{t}}E{\left(n\right)}_{dissipate\_t}\right)/N_{t}$$ (4)

where *N*_*t*_(≠0) is the number of living CRSNs nodes in round *t*; *E*(*n*)_*dissipate*_*t*_ is the energy consumed by control information exchange, data transmission and reception for node *n* in round *t*, and it depends on node identity and activity.

$$STD\left(E_{residual\_t}\right)=\sqrt{\frac{1}{N_{t}}\sum_{n=1}^{N_{t}}{\left(E{\left(n\right)}_{residual\_t}-AVG\left(E_{residual\_t}\right)\right)}^{2}}$$ (5)

where *E*(*n*)_*residual*_*t*_ is the residual energy of node *n* at the end of round *t*, and it is obtained by subtracting *E*(*n*)_*dissipate*_*t*_ from *E*(*n*)_*residual*_*t*-1_. It is noted that *E*(*n*)_*residual*_0_ = *E*_0_, here *E*_0_ is the initial energy of each CRSNs node when it is fully charged.

### 3.3 Periodical frame structure design of TD-IMOCRP

**Fig 3:**
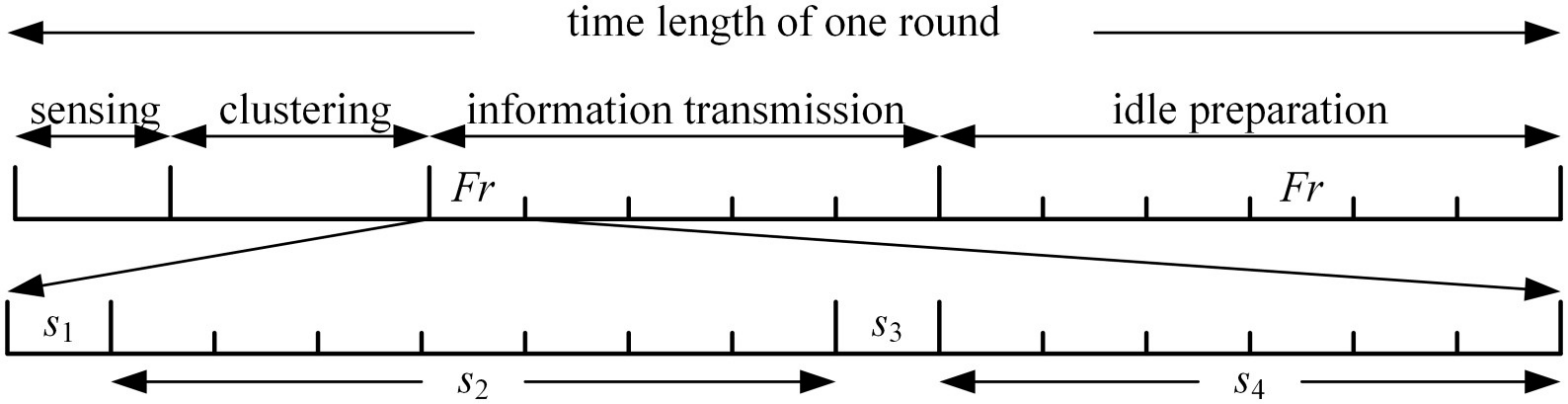
Periodical round structure of TD-IMOCRP.

Each round is composed of 4 phases, that is, spectrum sensing phase, clustering phase, information transmission phase and idle preparation phase. The periodical round structure is given in Fig 3. As event-driven traffic may occur at any time, in order to guarantee the in-time delivery of emergent event information, TD-IMOCRP adopts the clustering structure built for time-triggered traffic, and it gives higher priority to event-driven traffic. Each CRSNs node follows *Fr* frame structure to control its information exchange and data transmission.

*Fr* frame contains 4 time periods, i.e., *s*_1_, *s*_2_, *s*_3_ and *s*_4_. The time duration of each period and the schedule operations performed during these periods are explained in details as below:

1. Time period *s*_1_ includes 1 time slot during which lower-level nodes send time-triggered or event-driven data to upper-level nodes. Its length is equal to the time required to transmit 1 data packet. Here, the lower-level node is a normal CM or a CH, and the corresponding upper-level node is its CH or the sink.
2. Time period *s*_2_ is used by CMs to send event discovery signal to their CH. *s*_2_ is composed of multiple time slots whose number is equal to the number of CMs in the largest cluster communicating on the same channel. In this case, each CM can have its own time slot to transmit the event discovery signal. The objective of sending this signal is to notify the corresponding CH about the occurrence of emergent events, and time-triggered data transmission in this cluster should give way to event-driven data delivery.
3. Time period *s*_3_ includes 1 time slot which is used by the CH to broadcast event detection signal within its cluster.
4. Time period *s*_4_ is used for control information exchange between CHs and the sink. The number of slots in *s*_4_ is equal to the maximum number of CHs running on the same channel plus 1. Among them, the last time slot is reserved for the sink to inform CHs about the sequences of aggregated data transmission. Each of other slots is used by a CH to transmit aggregation completion signal (for both traffic types) or event detection signal (for event-driven traffic).

Above design is mainly for guaranteeing higher priority of event-driven traffic, in other words, it aims at avoiding cases in which event-driven data cannot be delivered timely if it occurs in the process of serving time-triggered traffic. The overall algorithm of TD-IMOCRP is shown in Algorithm 1 below.

**Algorithm 1**: TD-IMOCRP operation

**1** // **Phase 1: spectrum sensing**

**2** The sink broadcasts start signal for clustering.

**3 for** *i* = 1:*N*_*t*_

**4**  **if** *E*(*i*)_residual_t_>0

**5**   node *i* senses available channels and sends *E*(*i*)_*residual*_*t*_, (*x*_*i*_,*y*_*i*_) and **Channel(*i*)** to the sink.

**6**  **end**

**7 end**

**8** // **Phase 2: clustering**

**9** The whole CRSNs is grouped into multiple clusters according to IMO and the clustering results are broadcast by the sink towards the whole network.

**10** // **Phase 3: information transmission**

**11** // for time-triggered traffic

**12 for** *i* = 1:*N*_*t*_

**13**  **if** *i*∈CM

**14**   *i* sends time-triggered data to its CH in *s*_1_ period of *Fr* frame.

**15**  **else**

**16**   *i* aggregates data and sends the aggregation completion signal to the sink in *s*_4_.

**17**   Following the schedule instruction, *i* transmits the aggregated data to the sink in *s*_1_.

**18**  **end**

**19 end**

**20** // for event-driven traffic

**21 for** *i* = 1:*N*_*t*_

**22**  **if** *i*∈CM

**23**   **if** *i* detects emergent events

**24**    *i* sends event discovery signal to its CH in corresponding time slot of *s*_2_.

**25**    *i* sends event-driven data to its CH in corresponding time slot of *s*_1_.

**26**   **else**

**27 if**    *i* receives the event detection signal from its CH

**28**     *i* stops delivering time-triggered data to the CH in *s*_1_.

**29**    **else**

**30**     *i* sends time-triggered data to its CH.

**31**    **end**

**32** **end**

**33**  **else**

**34**   *i* broadcasts event detection signal to its CMs in *s*_3_.

**35**   *i* also sends event detection signal to the sink in corresponding time slot of *s*_4_.

**36**   *i* delivers aggregation completion signal and event-driven aggregated data to the sink in *s*_4_ and *s*_1_, respectively.

**37**  **end**

**38 end**

**39**// **Phase 4: idle preparation**

**40 if** event data has not been totally delivered to the sink at the beginning of next round

**41**  The event data transmission continues through previous clustering structure.

**42 else**

**43**  Time-triggered traffic will be served in next round.

**44 end**

Lines 1~7 in Algorithm 1 show the spectrum sensing phase. The sink broadcasts the start signal for clustering. After receiving the signal, each living CRSNs node performs spectrum sensing and sends the information about its residual energy *E*(*i*)_*residual*_*t*_, geographical location (*x*_*i*_,*y*_*i*_) and available channel list **Channel(*i*)** to the sink.

Lines 8~9 demonstrate the clustering phase. The sink determines node identity by using IMO algorithm, and then it informs each CRSNs node about the clustering results. The whole process is the same as that in IMOCRP, please refer to [6] for details.

**Fig 4:**
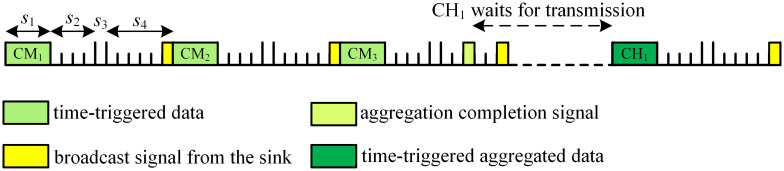
Transmission schedule of serving time-triggered traffic.

Lines 10~38 exhibit the detailed process of information transmission and we use a simple example to explain it. Assuming that cluster 1 has 1 CH and 3 CMs, i.e., CH_1_, CM_1_, CM_2_ and CM_3_. If cluster 1 only serves time-triggered traffic, its transmission schedule is shown in Fig 4. CM_1_ utilizes *s*_1_ in the first frame to send time-triggered data to CH_1_, and CM_2_ utilizes *s*_1_ in the second frame to perform the same function and so on. *s*_2_ and *s*_3_ periods are reserved for emergent events, therefore, no information is sent during these periods. After CH_1_ aggregates all time-triggered data from its CMs, it sends aggregation completion signal to the sink in *s*_4_. According to the schedule instruction broadcast by the sink, CH_1_ transmits the aggregated time-triggered data to the sink through *s*_1_.

**Fig 5:**
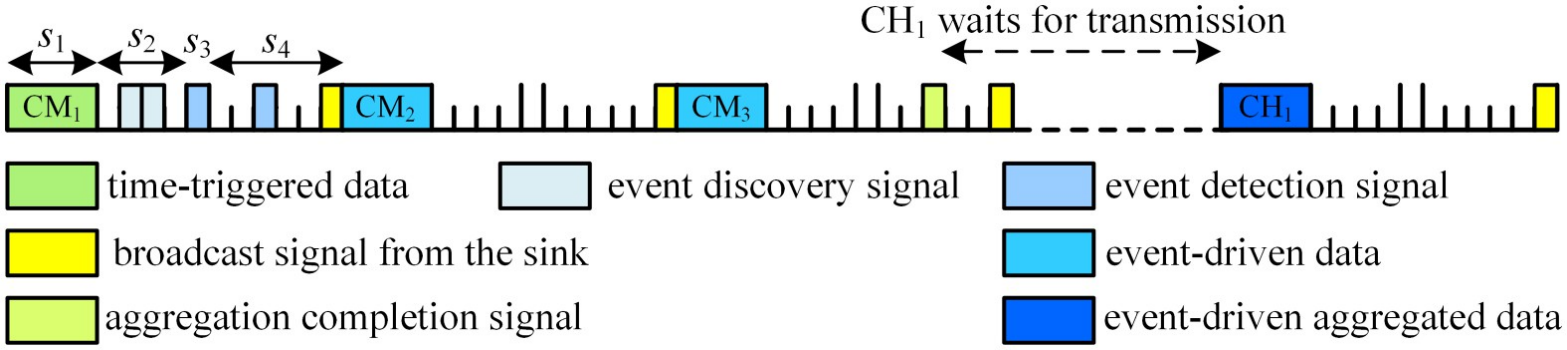
Transmission schedule of serving event-driven traffic.

When event-driven traffic occurs, CRSNs nodes who detect the emergent event follow the *Fr* frame structure to send event data to the sink immediately. Assuming that CM_2_ and CM_3_ detect emergent events in the first *Fr* frame, detailed schedule process is shown in Fig 5. CM_2_ and CM_3_ send event discovery signal to CH_1_ in corresponding time slots in *s*_2_. CH_1_ broadcasts event detection signal to its members in *s*_3_ with the purpose of informing them about the sequences of transmitting event data. On receiving the signal, all nodes in the cluster stop sending time-triggered data to the CH. The CH also sends the event detection signal to the sink in *s*_4_. CM_2_ and CM_3_ send event data to CH_1_ in corresponding time slots of *s*_1_ period. After data reception and aggregation, CH_1_ delivers completion signal to the sink in corresponding slot of *s*_4_ period. Intra-cluster data transmission in other clusters will not be affected by cluster 1, but according to the schedule information broadcast by the sink, cluster 1 will transmit event-driven aggregated data in priority. Therefore, time-triggered information transmission of other clusters on the same channel should be delayed.

**Fig 6:**
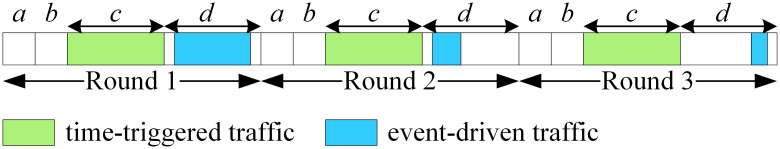
Periodical structure of TD-IMOCRP if event-driven data transmission can be finished in one-round period.

**Fig 7:**
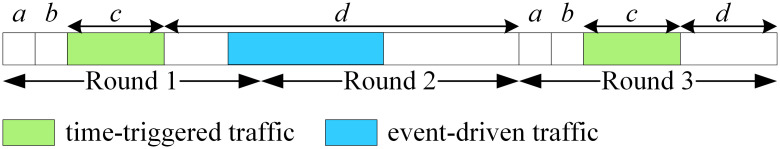
Periodical structure of TD-IMOCRP for cross-round event-driven data transmission.

Lines 39~44 exhibit the idle preparation phase. After serving time-triggered traffic, CRSNs nodes continue to detect events in this phase. It adopts the same information transmission manners as in phase 3. Due to the randomness of event-driven traffic, if certain CM detects events in this phase, 2 cases may happen:

1. The first case is shown in Fig 6. If CRSNs nodes can finish event-driven data transmission in this round, time-triggered traffic is normally served in next round. a, b, c, d represent spectrum sensing, clustering, information transmission and idle preparation phases, respectively.
2. The second case is shown in Fig 7. If CRSNs nodes detect events, but event data cannot be totally delivered to the sink in this round, the sink will not broadcast start signal for clustering at the beginning of next round. Event-driven data transmission continues by leveraging the clustering structure built in this round, and this transmission can last for 2 rounds at most. Otherwise, it is considered that transmission failure caused by dynamic access of PUs occurs. Therefore, time-triggered traffic is normally served in the third round.

## 4. Results and discussion

In this section, we use MATLAB to simulate our TD-IMOCRP and verify its effectiveness. As stated above, the network area is an 100m×100m square, and the sink is located at the center (or at position (0,0)). The period of time-triggered traffic is 0.5s. Event-driven traffic occurs every 20s, and it follows exponential distribution. The event radius is 30m, which means that CRSNs nodes within 30m around the event occurrence place can detect the event. In order to evaluate its performance in serving time-triggered traffic, we compare TD-IMOCRP with IMOCRP [6], as IMOCRP performs the best among current time-triggered clustering protocols for CRSNs. In addition, by comparing TD-IMOCRP with current event-driven clustering protocols such as ESAC [24], mESAC [25] and ERP [26], we test its capability in serving event-driven traffic.

### 4.1 Performance analysis of serving time-triggered traffic

**Fig 8:**
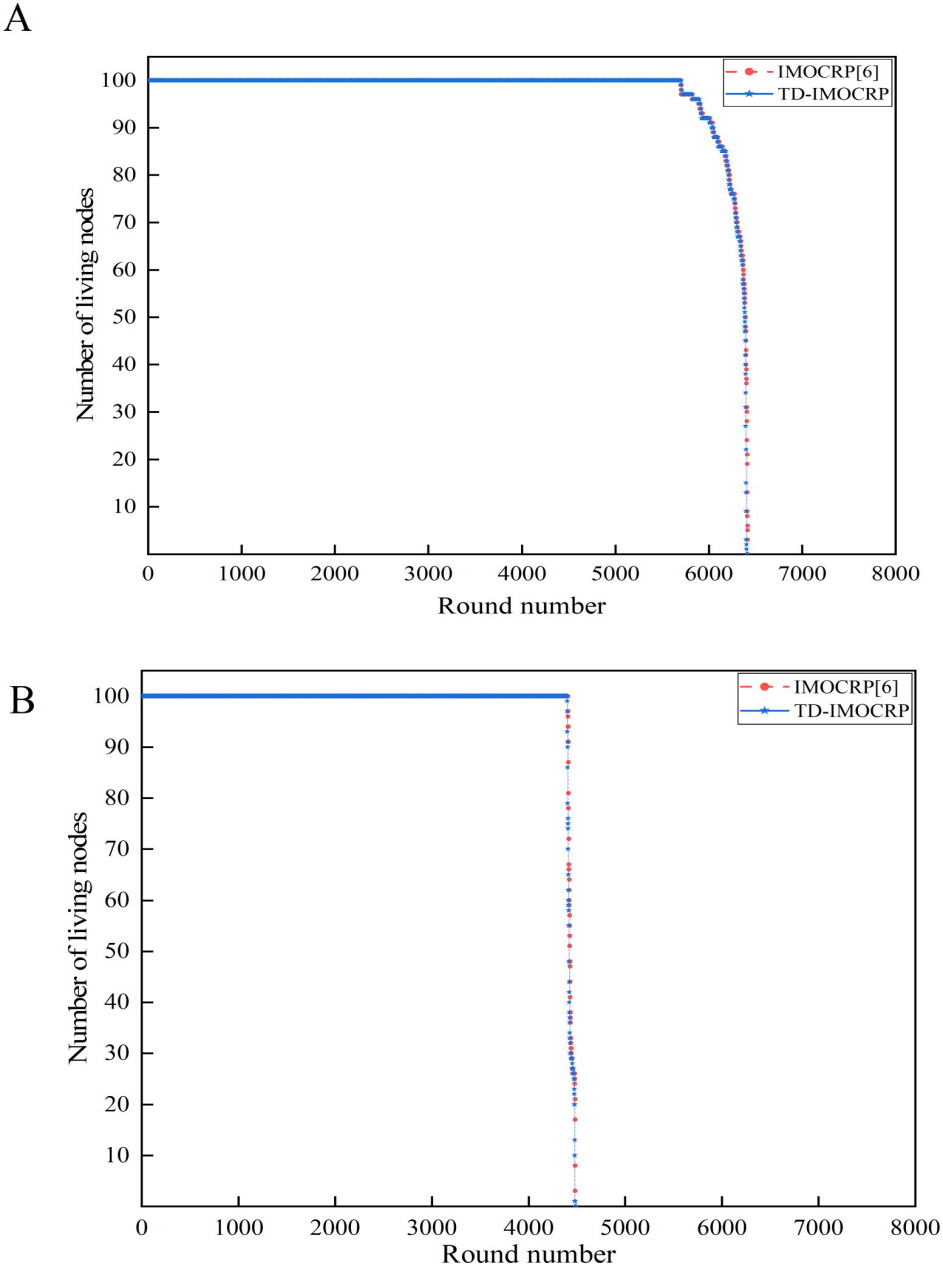
Comparison results of number of living nodes. (A) The sink is located at the center. (B) The sink is located at (0,0).

**Fig 9:**
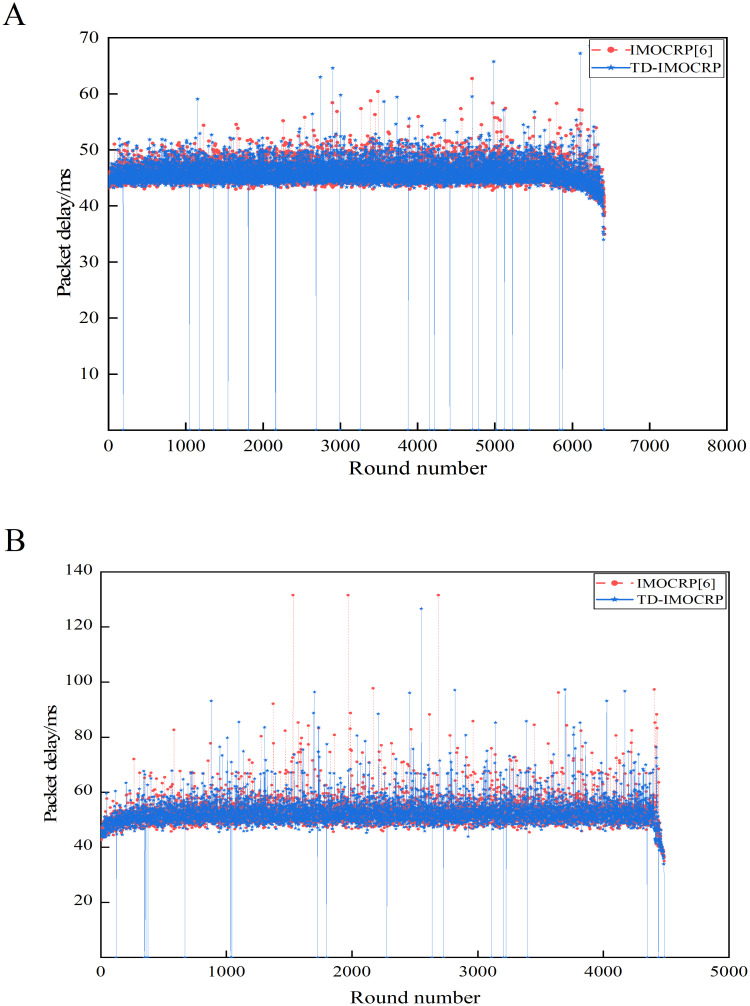
Comparison results of data packet delay. (A) The sink is located at the center. (B) The sink is located at (0,0).

As a representative time-triggered clustering protocol for CRSNs, IMOCRP exhibits good behaviors in reducing energy consumption, prolonging network lifetime and decreasing data packet delay. Therefore, IMOCRP is used as benchmark to evaluate the capability of TD-IMOCRP in serving time-triggered traffic. Performance evaluation is conducted under 2 network topologies, that is, the sink is located at the center and position (0,0), respectively. Number of living nodes and data packet delay are shown in Figs 8 and 9, respectively.

From Fig 8, the rounds in which the first node dies and the last node dies are shown in Table 2.

**Table 2:** Comparison results of network lifetime.

Protocols	Sink position	Round number	
		The first node dies	The last node dies
TD-IMOCRP	at the center	5707	6404
IMOCRP [6]		5701	6420
TD-IMOCRP	at (0,0)	4401	4483
IMOCRP [6]		4409	4486

From above simulation results, we can see that the first death node and the last death node of TD-IMOCRP appear a little earlier than IMOCRP. TD-IMOCRP serves both traffic types, which consumes more energy. Due to the low frequency of event-driven traffic which will not greatly influence node energy consumption, their difference in network lifetime is so small (several rounds) to be negligible. TD-IMOCRP is designed on the basis of IMOCRP, which means that they process time-triggered traffic with totally the same algorithm. To be specific, IMO algorithm is leveraged by the sink to determine CHs and their members based on the information received from all living CRSNs nodes. Each node follows the clustering results broadcast by the sink and performs its own tasks. Therefore, for TD-IMOCRP and IMOCRP, the energy consumption of serving periodical time-triggered traffic is almost the same. Compared with IMOCRP, TD-IMOCRP can also serve event-driven traffic which consumes extra energy. However, low frequency of event-driven information transmission has a very small impact on node energy consumption.

From Fig 9, we can observe that TD-IMOCRP and IMOCRP achieve similar performance in terms of time-triggered data packet delay as they run the same IMO algorithm. In addition, there are several rounds in which packet delay of TD-IMOCRP reaches 0, which means that time-triggered traffic cannot be served in these rounds. The reason is: as specified by TD-IMOCRP, event-driven information delivery can last for 2 rounds at most, that is, if event-driven data transmission cannot be finished within 1 round period, it will continue to this round. In other words, when event-driven traffic has not been served out, low-priority time-triggered data cannot be transmitted at all. Therefore, the data packet delay of time-triggered traffic in this round is 0. This is the cost paid for prioritizing event-driven data transmission.

According to the analysis above, TD-IMOCRP and IMOCRP have similar performance in serving time-triggered traffic. As we have verified in our previous work [6], IMOCRP performs better than majority of current time-triggered clustering protocols for CRSNs. Therefore, we can obtain the conclusion that TD-IMOCRP is superior to majority of current time-triggered clustering protocols for CRSNs in serving time-triggered traffic. However, due to high priority of event-driven traffic, time-triggered traffic in several rounds will be negatively affected.

### 4.2 Performance analysis of serving event-driven traffic

**Fig 10:**
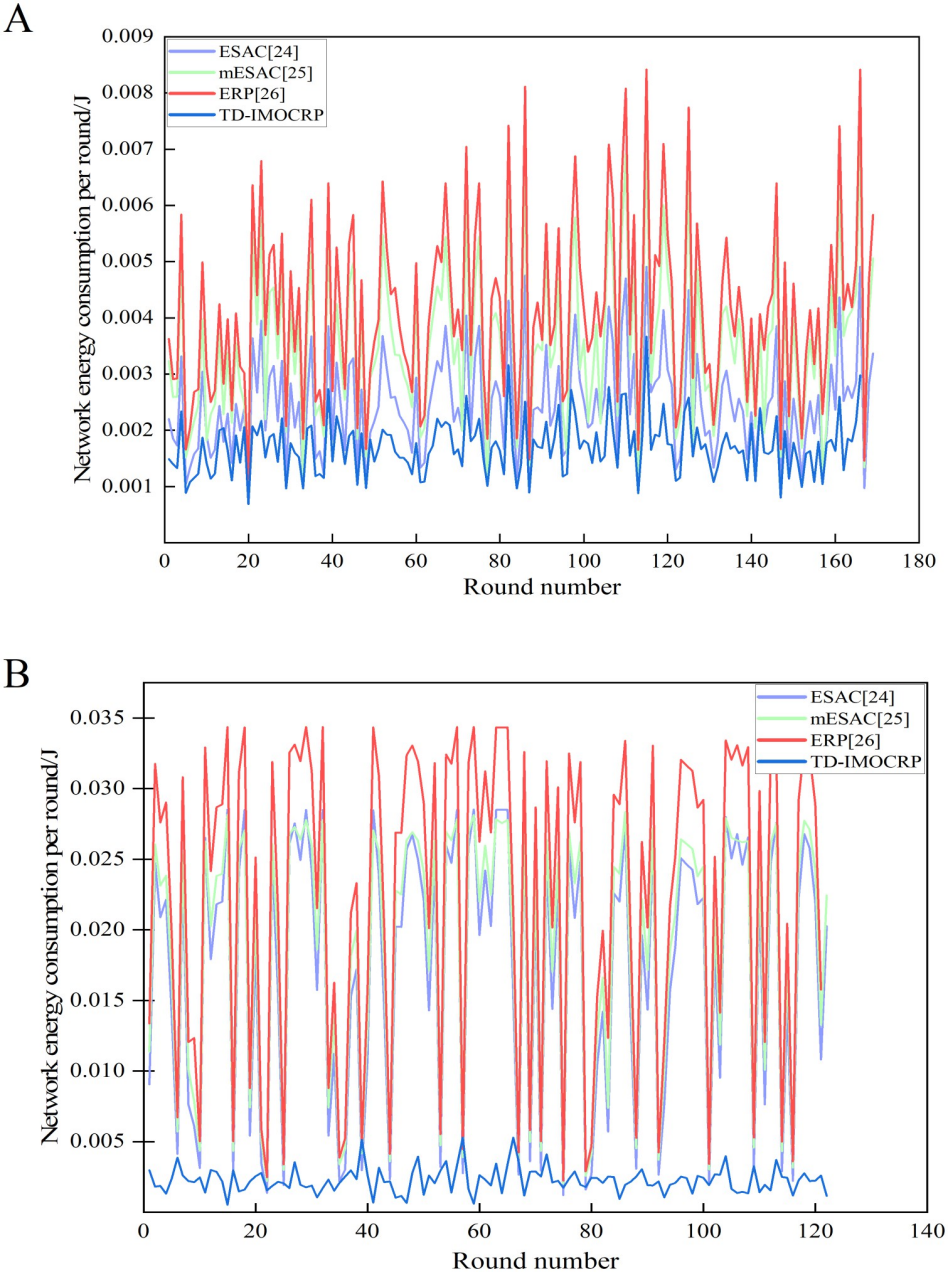
Comparison results of network energy consumption per round. (A) The sink is located at the center. (B) The sink is located at (0,0).

In this section, by comparing with ESAC [24], mESAC [25] and ERP [26], we will test and analyze the advantages of TD-IMOCRP in serving event-driven traffic. When the sink is located at the center and position (0,0), network energy consumption in each round is shown in Fig 10.

**Fig 11:**
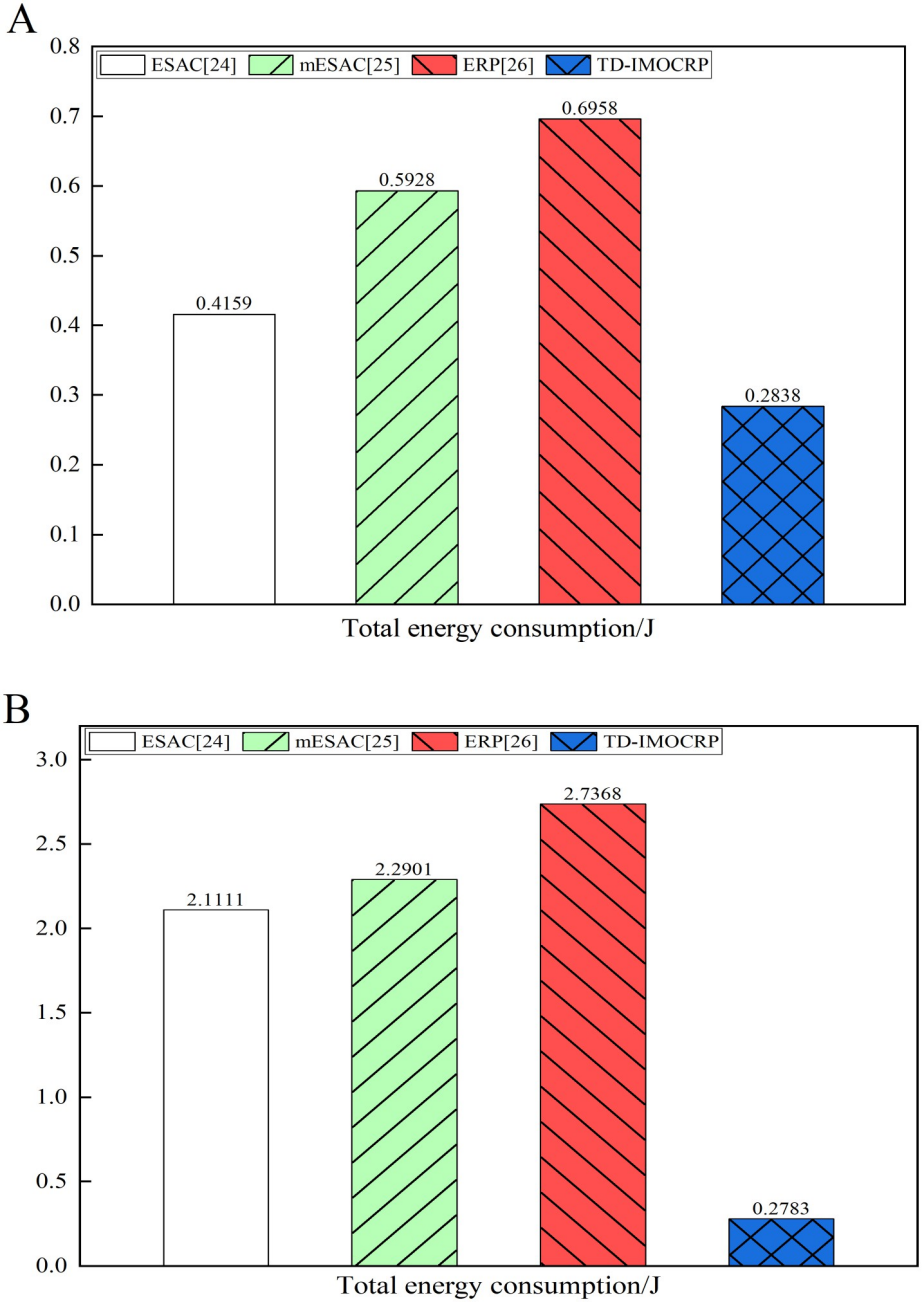
Comparison results of total energy consumption. (A) The sink is located at the center. (B) The sink is located at (0,0).

From Fig 10, we can observe that when serving event-driven traffic, network energy consumption of TD-IMOCRP is obviously lower than that of ESAC, mESAC and ERP in majority of rounds. In order to show its advantages more explicitly, we list the total energy consumption of each competing protocol in Fig 11 below.

**Fig 12:**
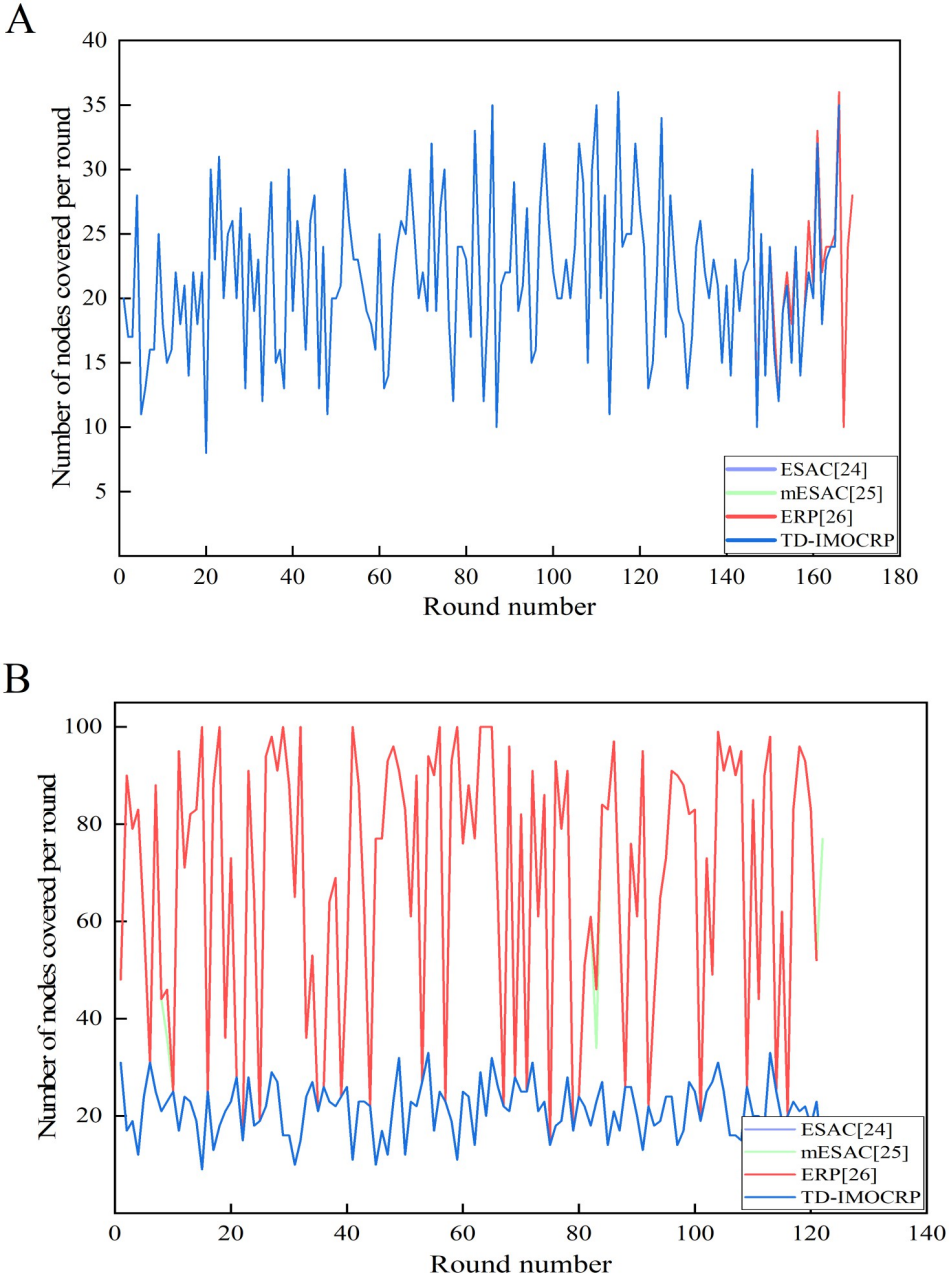
Comparison results of number of covered nodes per round. (A) The sink is located at the center. (B) The sink is located at (0,0).

**Fig 13:**
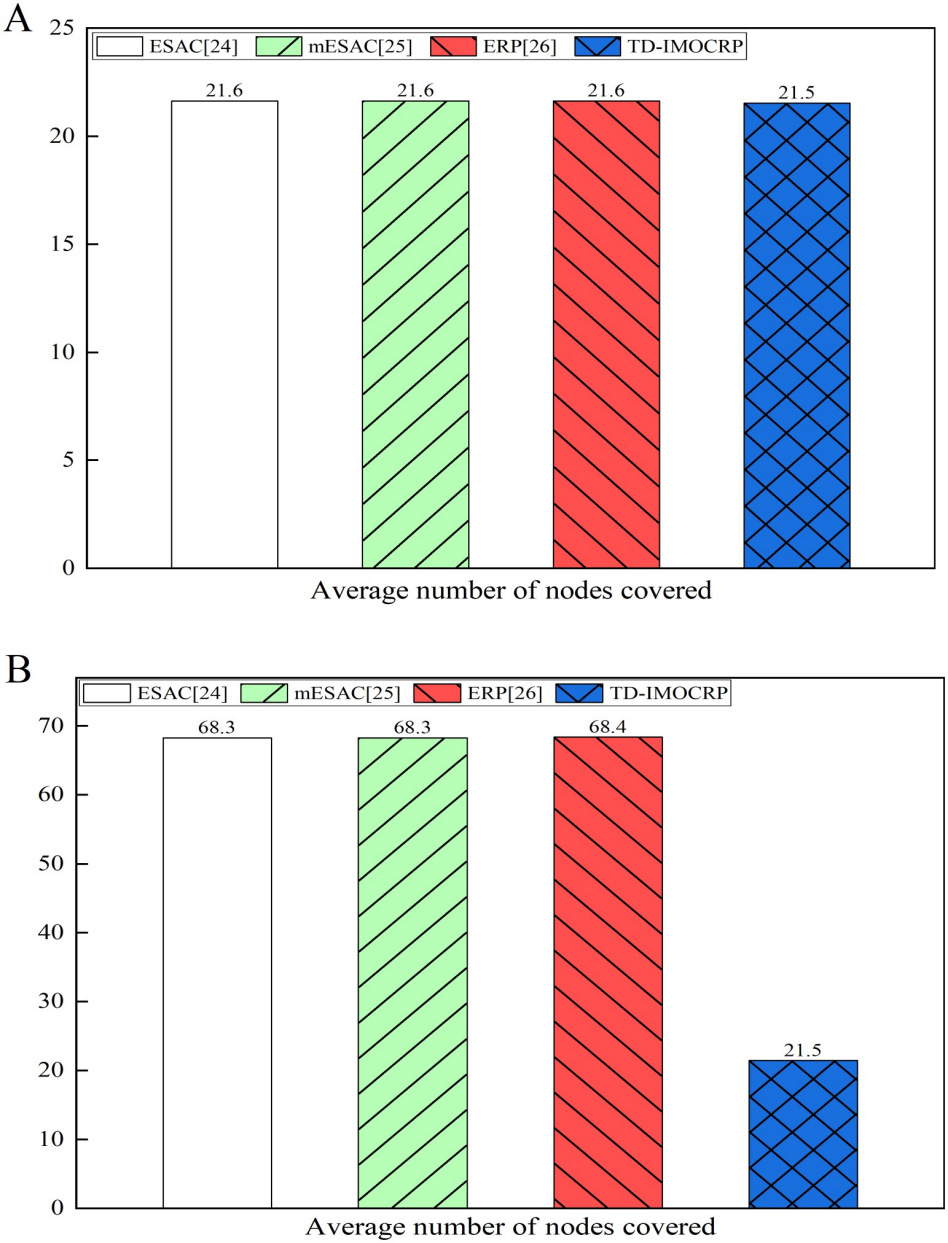
Comparison results of average number of covered nodes. (A) The sink is located at the center. (B) The sink is located at (0,0).

From Fig 11A, we can see that when the sink is located at the center, the total energy consumption of TD-IMOCRP is about 3/4 of that of ESAC, 1/2 of mESAC and 3/7 of ERP. As shown in Fig 11B, when the sink is located at (0,0), the total energy consumption of TD-IMOCRP is also much lower than that of other 3 protocols. The reasons lie in 2 aspects: (1) TD-IMOCRP directly uses the clustering structure built for time-triggered traffic to transmit event-driven information, therefore, CRSNs nodes do not need to consume extra energy for cluster construction after emergent event occurs, which helps conserve energy; Other 3 competing protocols determine eligibility corridor between emergent events and the sink through exchanging eligibility requests and responses in locality. In addition, cluster formation requests and responses are exchanged among neighboring eligible nodes to select CHs and form clusters. Transmission and reception of these control information consumes a huge amount of node energy. As for ERP, information is also required to be exchanged after cluster formation to help select primary and secondary gateways for inter-cluster packet relay. Therefore, its energy consumption is usually the highest among all competing protocols. (2) Number of CRSNs nodes which are covered by event-driven data transmission in TD-IMOCRP is much fewer than that of other 3 protocols. In TD-IMOCRP, CRSNs nodes which discover emergent events send corresponding information to their CH, and the CH forwards the information through inter-cluster route established for time-triggered information delivery. Other nodes will not be included in event-driven information transmission. In other 3 competing protocols, all CRSNs node located between the emergent events and the sink are judged to decide whether they satisfy corresponding eligibility requirements, such as closer to the sink, farther away from the events and so on. Eligible nodes form clusters to transmit the event-driven information to the sink. Therefore, more nodes are covered. In order to verify these factors, we record the number of nodes covered by event data transmission in each round and the average number of covered nodes, and the results are given in Figs 12 and 13.

From Figs 12A and 13A, we can observe that when the sink is located at the center, the curve of number of nodes covered per round by each protocol is overlapped with each other. Of course, ERP covers a slightly higher number of nodes than other competing protocols in certain rounds. Small distance between the event area and the sink will not introduce many candidate valid nodes. Correspondingly, the number of valid nodes covered by event data transmission in other protocols is basically the same as that of TD-IMOCRP. From average point of view, the number of nodes covered is over 20. However, when the sink is located at (0,0), as shown in Figs 12B and 13B, there is a big gap in the number of nodes covered by TD-IMOCRP and other protocols, while other competing protocols achieve almost the same performance. As stated above, in ESAC, mESAC and ERP, all living nodes which are located between event area and the sink are considered as valid nodes, and they all participate in transmitting event data. For these 3 protocols, when the sink is located at the corner, the large distance between the event area and the sink results in an increasing number of valid nodes. TD-IMOCRP do not need to select valid nodes by judging their position. Instead, CMs which detect the event directly send event data to the sink through the relay of their CH, and this helps reduce the number of nodes participating in event-driven information transmission. Compared with ESAC, mESAC and ERP, TD-IMOCRP can decrease the number of covered nodes by about 66.3% on average. From above results, we can conclude that TD-IMOCRP is more powerful in adapting to different network topologies.

**Fig 14:**
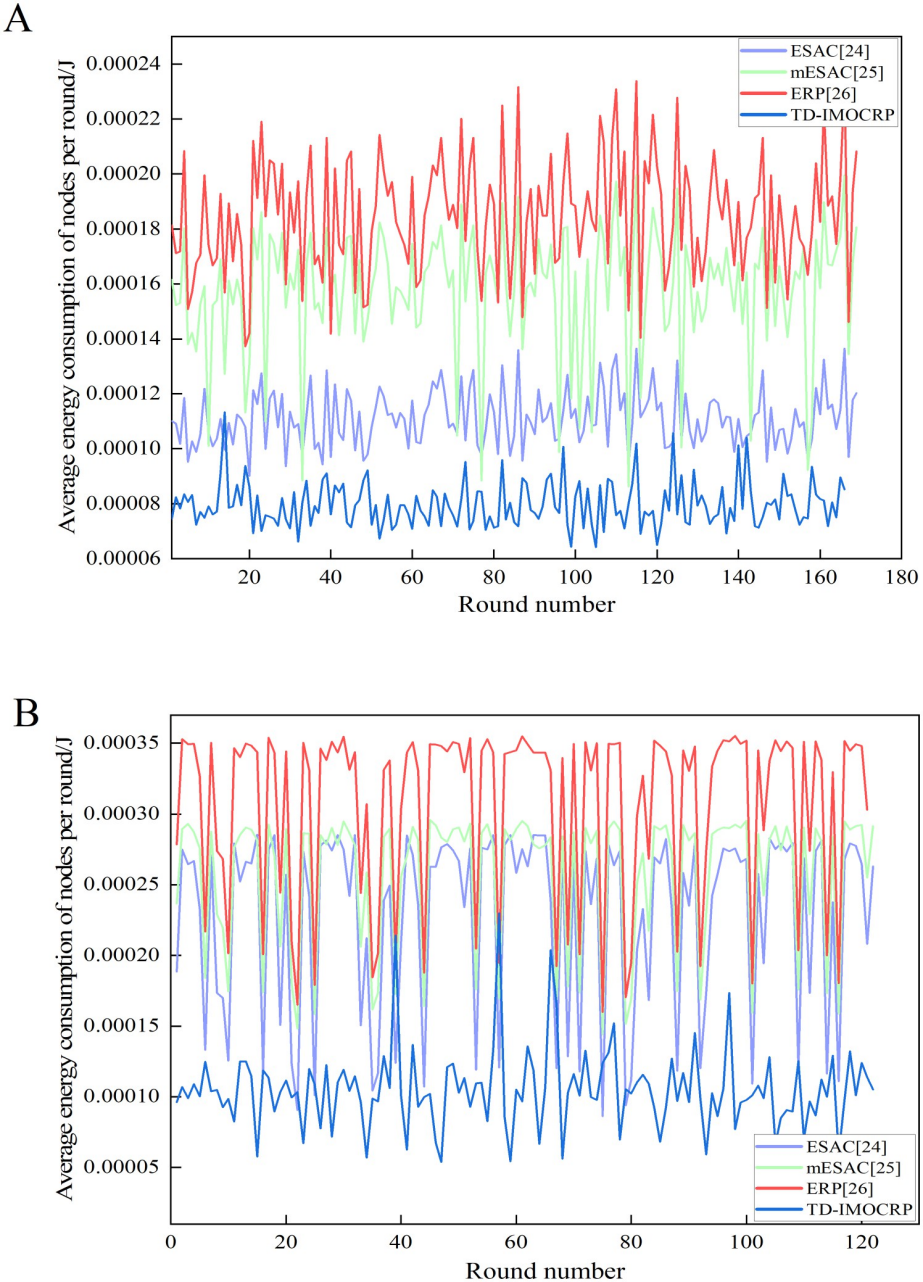
Comparison results of average energy consumption of nodes per round. (A) The sink is located at the center. (B) The sink is located at (0,0).

Apart from the number of covered nodes, we also calculate the average energy consumption of nodes in each round under 2 topologies, i.e., the sink is located at the center and the corner, and the results are shown in Fig 14.

From Fig 14, we can observe that when serving event-driven traffic, the average energy consumption of nodes in TD-IMOCRP is still significantly lower than that of ESAC, mESAC and ERP. To be specific, the average energy consumption of CRSNs nodes in TD-IMOCRP is about 2/15 of that of ESAC, 1/8 of mESAC and 1/10 of ERP. The reason is that when serving event-driven traffic, CRSNs nodes in TD-IMOCRP only consume energy to transmit data, and there is no energy consumed by cluster construction and route establishment after emergent events, which helps achieve lower energy consumption. As for other 3 competing protocols, their average number of covered nodes is almost the same, therefore, the average energy consumption of nodes per round exhibits similar trend as the network energy consumption per round, i.e., ERP is the highest, followed by mESAC and finally ESAC. Above simulation results all demonstrate that TD-IMOCRP shows excellent properties in reducing energy consumption and prolonging network lifetime. As for data packet delay, as TD-IMOCRP does not include determining identity of data transmission nodes, selecting CHs/CMs, data packet delay can be reduced, which improves the timeliness of event information transmission.

In a word, TD-IMOCRP can serve both time-triggered traffic and event-driven traffic effectively. It gains obvious advantages over existing event-driven clustering protocols in requiring fewer nodes to participate in emergent event information transmission, reducing node energy consumption and shortening information transmission delay. In addition, it achieves almost the same performance as IMOCRP in terms of network lifetime and data packet delay when serving time-triggered traffic. However, the time-triggered traffic in several rounds may not be served due to higher priority of event-driven traffic. In other words, TD-IMOCRP dramatically improves its performance in serving event-driven traffic at the cost of slightly compromising its performance in serving time-triggered traffic.

## 5. Conclusions

In this paper, a traffic-driven clustering protocol for CRSNs is proposed. Based on IMOCRP, TD-IMOCRP periodically groups the whole CRSNs into clusters and collects surveillance information. It can achieve similar performance as IMOCRP which is better than majority of current time-triggered clustering protocols for CRSNs. If emergent event occurs, thanks to its special *Fr* frame structure, TD-IMOCRP can serve event-driven traffic immediately. Compared with traditional event-driven clustering protocols for CRSNs, TD-IMOCRP can decrease data packet delay, as clustering structure has already been built before event occurrence. In addition, as illustrated in Figs 12 and 13, the number of nodes covered by TD-IMOCRP is lower than its competing protocols, especially when the sink is located at the corner of the network. TD-IMOCRP leverages the clustering architecture built for time-triggered traffic to transmit event-driven information. Only the CRSNs nodes which discover emergent events and corresponding CHs participate in data transmission. In contrast, in ESAC, mESAC and ERP, all CRSNs nodes located between emergent events and the sink participate in cluster construction and data forwarding. More involved nodes mean higher energy consumption. Therefore, TD-IMOCRP has the advantage of reducing network energy consumption. However, as TD-IMOCRP requires direct information exchange between CRSNs nodes and the sink before clustering, it is assumed that each CRSNs node can reach the sink through single-hop communication, which restricts network scalability. Therefore, it cannot be applied to large-scale CRSNs. In our future work, we will further study multi-hop information transmission between CRSN nodes and the sink to extend the application scope of our TD-IMOCRP.

## Supporting information

S1 File This file contains a dataset in EXCEL format (XLSX) used to plot Figs 8 to 14. (XLSX)
